# Pre-Exposure to Environmental Enrichment Protects against Learning and Memory Deficits Caused by Infrasound Exposure

**DOI:** 10.1155/2022/6208872

**Published:** 2022-05-17

**Authors:** Shan Jiang, Yong-Qiang Wang, Yi-Fei Tang, Xi Lu, Dan Guo

**Affiliations:** ^1^The China-Japan Friendship Hospital, Beijing 100029, China; ^2^Sunshine Union Hospital, Weifang Shandong 261071, China

## Abstract

With the development of industrialization in recent years, infrasound has become an important component of public noise. To date, diverse studies have revealed the negative effects of infrasound on the central nervous system (CNS), especially the learning and memory ability. It is widely reported that environmental enrichment (EE) ameliorates the learning and memory deficits in different models of brain injury. Therefore, the present study was designed to determine the possible benefits of pre-exposure to EE in preventing functional deficits following infrasound exposure and their related mechanism. Adult male rats were given enriched or standard housing for 30 days. Following enrichment, the rats were exposed to 16 Hz, 130 dB infrasound for 14 days, and then their learning and memory ability was assessed. Changes to neuroinflammation, apoptosis, and oxidative stress in the hippocampus were also detected. Our results showed that the infrasound-induced deficit in learning and memory was attenuated significantly in EE pre-exposed rats. Pre-exposure to EE could induce a decrease in proinflammatory cytokines and increased anti-inflammatory cytokines and antioxidant properties in the hippocampus. Moreover, pre-exposure to EE also exerted antiapoptosis functions by upregulating the B-cell lymphoma/leukemia-2 (Bcl-2) level and downregulating the P53 level in the hippocampus. In conclusion, the results of the present study suggested that EE is neuroprotective when applied before infrasound exposure, resulting in an improved learning and memory ability by enhancing antioxidant, anti-inflammatory, and antiapoptosis capacities.

## 1. Introduction

Infrasonic noise refers to acoustic oscillation with a frequency below 20 Hz, which is hard to detect by the human ear [1]. There are many natural sources of infrasonic noise, including volcanic eruptions, ocean waves, and wind [2]. Currently, modern society has greatly increased infrasound generation through man-made sources, such as occupational conditions, industrial installations, vibration of mechanical equipment inside enclosed spaces, wind turbines, and transportation [3]. Opening the rear window in a car traveling at 100 km/h for example exposes the passengers to levels of infrasound as high as 125 dB [4]. Its characteristics of strong vibration/penetration, low attenuation during long distance propagation, and difficulty in protection mean that infrasound has become an important component of noise pollution and a new health hazard to the public at large [5].

Mammalian organs' inherent vibration frequencies are just within the range of those of infrasound; therefore, infrasound can disturb normal functions of multiple organs by triggering biological resonance [6, 7], in which the central nervous system (CNS) is the most vulnerable organ [8–10]. Substantial and growing evidence has revealed that exposure to infrasound can markedly impair the learning and memory ability of rats [9, 11]. The underlying mechanisms include enhanced neuronal apoptosis, production of proinflammatory cytokines, oxidative stress, and microglial activation in the rat hippocampus [11–13]. However, it is difficult to prevent human beings from infrasound-induced learning and memory deficit because it is detectable in most cases [14]. Thus, it is necessary to explore novel methods that effectively prevent against infrasound-induced learning and memory deficit.

Environmental enrichment (EE) is a paradigm consisting of enriched and novel housing conditions. EE has been reported to be capable of ameliorating cognitive function deficits associated with various brain injuries [15–17]. For example, EE has shown its potential protective effects against memory deficits in a rat model of traumatic brain injury (TBI) [18–20]. This effect is achieved by decreasing the level of proinflammatory cytokines (interleukin- (IL-) 1*β* and tumor necrosis factor alpha (TNF*α*)) and increasing the level of the anti-inflammatory cytokine (e.g., IL-10) [18–20]. In a rat stroke model, EE was also shown to prevent stroke-induced learning and memory disorder [21–23]. In this process, EE alleviated oxidative stress, suppressed neuroinflammation, reduced cytokines, and alleviated astroglial activation [21–23]. Our recent research also showed that EE can protect against sepsis-associated encephalopathy- (SAE-) induced learning and memory deficits by decreasing the cytokines in the hippocampus and this effect was mediated by vasopressin (VP) binding to the VP receptor 1a [24]. Although EE therapy is effective in reducing negative outcomes, its efficacy is limited by the fact that the damage has already occurred, and its usage is palliative rather than preventative. In recent years, a growing body of research has focused on the preventive effect of EE exposure before injury. Johnson and colleagues demonstrated that preinjury exposure to EE enhanced resistance against cognitive deficits caused by TBI [25]. In an experimental model of cerebral ischemia, exposure to EE before cerebral ischemia induction also exhibited a cognitive neuroprotective effect [26]. Further research showed that pre-exposure to EE can reduce the level of the inflammatory cytokines and relieve the oxidative damage that contributes to cognitive impairment [26, 27]. All these findings suggest that pre-exposure to EE might be capable of generating tolerance against infrasound-induced learning and memory impairment. However, little information is available in the literature regarding the protective effects of pre-exposure to EE against infrasound-induced learning and memory impairment.

Accordingly, the aim of the present study was to evaluate the preventive effect of pre-exposure to EE on infrasound-induced learning and memory impairment and its underlying mechanism. To this end, rats were given enriched or standard housing for 30 days, and then exposed to 16 Hz, 130 dB infrasound for 14 days. Their learning and memory abilities were then evaluated. Additionally, we detected the changes in neuroinflammation, apoptosis, and oxidative stress in the hippocampus. If it demonstrates a protective effect on learning and memory ability, pre-exposure to EE could be an effective method to protect against learning and memory deficits after infrasound pollution and could be applied in clinical practice.

## 2. Materials and Methods

### 2.1. Animals

Male Sprague-Dawley (SD) rats (200–250 g) were provided by the Animal Center of the China-Japan Friendship Hospital, Beijing, China. The experimental procedures were carried out in accordance with the *Guidelines for the use of animals in neuroscience research* (published in the Membership Directory of the Society, pp 27-28, 1992) and were approved by the committee of Animal Use for Research and Education of the China-Japan Friendship Hospital. The animals were housed under a 12 h light/dark cycle in a temperature-controlled room at 24 ± 1°C with free access to food and water. In addition, animals were allowed one week of acclimation to the experimental room before the experiments.

### 2.2. Housing Conditions

Two housing conditions were used in this study: EE conditions and standard environment conditions (SE).

SE: Rats were housed in standard-sized polycarbonate cages (25 cm ×40 cm ×20 cm), with two rats per cage located in a quiet room. The cages allowed for moderate activity and exploration.

EE: Rats were housed in a large cage (40 cm ×54 cm ×30 cm) with six rats per cage. As previously described, the cages contained multiple objects including wooden blocks, plastic bone-shaped toys, a running wheel, and a plastic tunnel [24]. Suspended ropes allowed for climbing from one level to another. Objects were replaced twice a week. During the enrichment period, food and water were available *ad libitum*. After 30 days of EE housing, the levels and toys were removed from the cages such that the animals no longer received additional enrichment.

### 2.3. Experimental Grouping

According to the results of preliminary experiments and previous studies [11, 28], the sample size for each experiment was determined.

**Figure 1:**
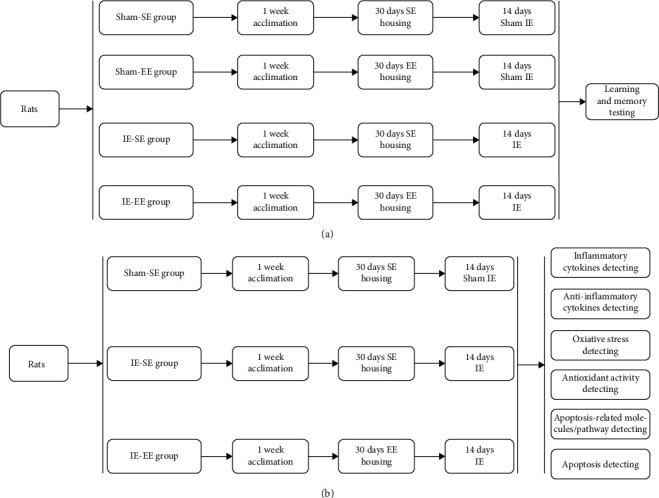
Experimental flow chart. (a) Learning and memory testing. (b) Mechanism experiments.

For learning and memory testing, 24 rats were assigned in equal numbers to four groups (6 rats in each group) (Figure 1(a)): (1) the Sham-SE group. In this group, the rats were maintained under SE conditions and were then placed in the infrasonic chamber for 2 h once daily for 14 days, but without infrasound exposure (IE) (i.e., sham IE); (2) the Sham-EE group. In this group, the rats were maintained under EE conditions and then received sham IE; (3) the IE-SE group. In this group, the rats were maintained under SE conditions, placed in the infrasonic chamber, and treated with 16 Hz and 130 dB IE for 2 h once daily for 14 days; (4) the IE-EE group. In this group, the rats were maintained under EE conditions and then placed in the infrasonic chamber for and treated with 16 Hz and 130 dB IE for 2 h once daily for 14 days. For more detailed information about infrasound device and its parameters, see Section 2.4.

**Figure 2:**
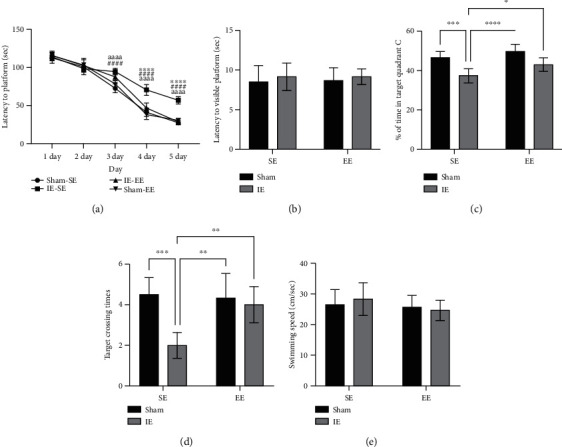
(a) From day 3 onwards, the rats in the IE-SE group showed a longer latency time to the platform than the rats in the Sham-SE, Sham-EE, or IE-EE group. Sham-SE *vs*. IE-SE: ^####^*p* < 0.0001; IE-SE *vs*. IE-EE: ^∗∗∗∗^*p* < 0.0001; IE-SE *vs*. Sham-EE: ^aaaa^*p* < 0.0001. (b) No differences were exhibited in the latency to the visible platform. (c) The rats in the IE-SE group spent less time in target quadrant C compared with the rats in the Sham-SE, Sham-EE, or IE-EE group. ^∗^*p* < 0.05, ^∗∗∗^*p* < 0.001, ^∗∗∗∗^*p* < 0.0001. (d) The rats in the IE-SE group had lower target crossing times compared with the rats in the Sham-SE, Sham-EE, or IE-EE group. ^∗∗^*p* < 0.01, ^∗∗∗^*p* < 0.001. (e) No differences were observed in the swimming speed. The assignment of order was counterbalanced across rats in this test. Data represent means ± SEM, *n* =6.

There were no differences in the outcomes of the Morris Water Maze (MWM) test between the Sham-EE group and Sham-SE group (see Section 3.1 and Figure 2); therefore, we only set a Sham-SE group as the control for the mechanism experiments (Figure 1(b)). Rats (*n* =54) were assigned in equal numbers to three groups for the mechanism experiments (18 rats in each group): the Sham-SE group, the IE-SE group, and the IE-EE group (Figure 1(b)). To investigate the mechanism of pre-exposure to EE, we detected the level of inflammatory/anti-inflammatory mediators, oxidative/antioxidant activity, apoptosis, and apoptosis-related molecules and pathway. Thus, in each group, 6 rats were used to detect the level of apoptosis and apoptosis-related molecules using Fluoro-Jade C (FJC) staining and immunofluorescent microscopy. The rats were sacrificed and brain sections containing the hippocampus were prepared for staining. Another 6 rats were used to detect the oxidative/antioxidant activity. The rats were sacrificed and the hippocampus was homogenized. Then, homogenates were tested for oxidant and antioxidant activity. Another 6 rats were used to detect the levels of inflammatory/anti-inflammatory mediators and apoptosis-related molecules using quantitative real-time reverse transcription PCR (qRT-PCR) and enzyme-linked immune-absorbent assays (ELISAs). The rats were sacrificed and their brains were divided into the two hemispheres. The hippocampus on one side was used for qRT-PCR and the hippocampus on the other side was used for ELISA.

### 2.4. Infrasound Device

After 30 days of housing (EE or SE), the rats were given infrasound treatment.

The infrasound device used in this study has been described in our previous study [7]. The infrasound device consists of an infrasound generator (1110B, Beijing Intensity Environment Institute, Beijing, China) with a power amplifier (No. 7101, Beijing 702 Institute of Spaceflight Co, Beijing, China), a chamber containing four loudspeakers (YD500-8XA, Nanjing Electroacoustic Equipment Co., Nanjing, China), an infrasonic sensor (ACO Pacific, Belmont, CA, USA), and a data collection system. The electric-actuated infrasound generator can generate infrasound of 16 Hz at 90–130 dB. A real-time ultra-low frequency signal acquisition system was used to collect and analyze the frequency and intensity of infrasound. The frequency and intensity of infrasound are monitored using an infrasonic sensor and displayed on the computer. The infrasonic generator system can generate standard infrasonic waves with a frequency range from 2 to 20 Hz and a sound pressure level from 90 to 140 dB. The intensity and frequency were held steady during 2 h of animal exposure and were monitored by the data collection system.

According to previous studies [7], the rats' learning and memory abilities were most seriously affected when exposed to 16 Hz at 130 dB infrasound, and thereby this parameter was adopted in the present study. The IE-SE or IE-EE groups were exposed to infrasound of 16 Hz and 130 dB for 2 h once daily for 14 days. The Sham-SE or Sham-EE group was placed into the chamber without infrasound exposure.

### 2.5. Learning and Memory Testing

The MWM is widely used to detect spatial learning and reference/working memory [29, 30]. This maze comprised a dark circular tank (178 cm in diameter) that was virtually divided into four quadrants (A, B, C, D). The tank was filled with water (approximately 37 cm deep). A plexiglass platform (10.2 cm in diameter) was submerged to a depth of 2 cm below waterline (i.e., invisible to the rat) and placed approximately 28 cm from the pool wall of quadrant C. To provide external space clues, several extra-maze visual objects of different shapes and sizes were hung on the wall of the experimental room. In this test, spatial learning was detected using a 5-day block comprising of four trials per day. For each trial, the rats were placed in the pool facing the wall at random quadrants. If the rats did not climb onto the platform in 120 s, they were physically guided to it. Once they reached the platform, the rats remained on it for 30 s and were then given a 5-minute break before the next trial. As a control, the platform was raised 2 cm above the water surface (visible to the rat) on day 6 to identify the contributions of non-spatial factors. On the same day, memory retention was also measured through a single probe trial. The platform was removed, and the rats explored the tank for 30 s. The percent time spent in the target quadrant (quadrant C) and the number of crossings of the platform's previous location were recorded.

### 2.6. Enzyme-Linked Immune-Absorbent Assay (ELISA)

Inflammatory mediators in the hippocampus were measured using ELISAs. Rats were killed and decapitated. Tissues were collected for ELISA as previously described [31]. Briefly, the brains were placed in a chilled matrix and microdissected on a chilled glass plate. The hippocampus was isolated, homogenized with normal saline, and centrifuged at 2000 rpm, 4°C for 10 min. The supernatants were used to detect interleukin IL-10, IL-6, IL-1*β*, TNF-*α*, BCL2 associated X, apoptosis regulator (BAX), and caspase-3 using ELISA kits (Nanjing Jiancheng Bioengineering Institute, Jiangsu, China) following the manufacturer's instructions.

### 2.7. Measurement of Oxidant Activity in the Hippocampus

#### 2.7.1. Lipid Peroxidation

To determine the extent of lipid peroxidation in hippocampal homogenates, thiobarbituric acid-reactive substances (TBARS) were determined using the method described by Mihara and Uchiyama [32], with minor modifications. Hippocampi were isolated and sonicated in 10% (w/v) using radioimmunoprecipitation assay (RIPA) buffer (Tris 50 mM pH 7.4, 1% Triton X-100, NaCl 150 mM, NaF 5 mM, 0.1% sodium dodecyl sulfate, and 1% sodium deoxycholate), to which a protease inhibitor cocktail (Sigma, St. Louis, MO, USA) was added. Homogenates were incubated on ice for 30 min and then centrifuged at 600 × *g* for 10 min (4°C). The supernatants were stored at -80°C until analysis. A malondialdehyde acid (MDA) standard curve was obtained by acid hydrolysis of tetraethoxypropane. The TBA-MDA reaction was carried out by incubation at 95°C for 10 min. Fluorescence was measured at an excitation wavelength of 515 nm and an emission wavelength of 548 nm.

#### 2.7.2. Advanced Oxidation Protein Products (AOPP)

Measuring AOPP directly determines the amounts of oxidized proteins in biological samples. For AOPP determination, tissue was homogenized by sonication in cold buffer containing 50 mM NaH_2_PO_4_ and 1 mM EDTA at pH 7.5. Then, homogenates were centrifuged at 10,000 × *g* for 10 min (4°C). Spectrophotometric determination of AOPP levels was performed at 340 nm according to Barsotti's method [33].

#### 2.7.3. Nitric Oxide (NO)

The final and stable end products of NO *in vivo* are nitrates and nitrites, the sum of which (NOx) reflects the total NO production. NOx was determined using a colorimetric assay (Cayman Chemical Company, Ann Arbor, MI, USA) as described previously [34]. The nitrates in the sample homogenates were enzymatically converted into nitrites by incubation with nitrate reductase and NADPH, and total nitrite (nmol/mg protein) were then monitored using the Griess reaction at 540 nm.

### 2.8. Measurement of Antioxidant Activity in the Hippocampus

#### 2.8.1. Glutathione System

Reduced glutathione (GSH) and glutathione disulfide (GSSG; oxidized glutathione) concentrations were measured in hippocampal extracts. A sample of tissue was homogenized in a cold 1 : 1 mixture of 0.1 M potassium phosphate, 5 mM EDTA (pH 6.8), and 10% metaphosphoric acid. The homogenate was incubated on ice for 30 min and then centrifuged at 100,000 × *g* for 30 min. The resulting supernatant was used to determine GSH and GSSG using the fluorescent probe ophthalaldehyde (OPA). Aliquots for GSSG determination were first incubated with N-ethylmaleimide, which complexes with GSH, to avoid interference. After a further 15 min of incubation with OPA, fluorescence was determined at 420 nm (excitation 350 nm). The total brain proteins were determined using the Bradford protein assay. For glutathione reductase (GR, EC 1.8.1.7) and glutathione peroxidase (GPx, EC 1.11.1.9) determinations, hippocampi were sonicated in cold buffer containing 50 mM NaH_2_PO_4_ and 1 mM EDTA at pH 7.5. Then, the homogenates were centrifuged at 10,000 × *g* for 10 min (4°C). The supernatant was used to determine either GR or GPx activity using a kit (Nanjing Jiancheng Bioengineering Institute) according to the manufacturer's instructions.

#### 2.8.2. Superoxide Dismutase

Hippocampal tissue was homogenized by sonication as described in Section 2.7. Then, homogenates were centrifuged at 600 × *g* for 10 min (4°C). The superoxide dismutase activity (SOD, EC 1.15.1.1.) was estimated from the supernatant using a kit (Nanjing Jiancheng Bioengineering Institute).

### 2.9. Immunofluorescent Microscopy

Rats were sacrificed by perfusion fixation, in which the animals were deeply anesthetized using an injection of sodium pentobarbital (40 mg/kg, i.p.) and perfused transcardially with 10 ml of saline, followed by 40 ml of phosphate buffer (PB; pH 7.4) containing 4% paraformaldehyde. The brains were removed immediately and placed in 0.1 M PB containing 30% sucrose overnight at 4°C. Next day, the brain samples containing the hippocampus were cut serially into coronal sections of 25 *μ*m thickness on a freezing microtome. These brain sections were mounted onto glass slides or collected in phosphate buffered saline (PBS, pH 7.4) for staining.

To identify B-cell lymphoma/leukemia-2 (Bcl-2) or p53 protein expression, mouse anti-p53 IgG (1 : 250, Abcam, Cambridge, MA, USA) and rabbit anti-Bcl-2 IgG antibodies (1 : 200, Abcam) were used. The negative control for every experiment was constructed by replacing the primary antibodies with 1% bovine serum albumin (BSA)-PBS. Immunofluorescence staining was performed on the hippocampus sections. Briefly, the sections were incubated for 48 h at 48°C with a mixture of primary antibodies in 0.01 M PBS (pH 7.4) containing 1% normal donkey serum, 3% BSA, and 0.1% Triton X-100. Subsequently, the sections were rinsed in 0.01 M PBS (pH 7.4), and then incubated with Alexa Fluor 488 conjugated donkey antimouse IgG (1 : 500; Molecular Probes, Eugene, OR, USA) or Alexa Fluor488 conjugated donkey antirabbit IgG (1 : 500; Molecular Probes) diluted in PBS for 4 h at room temperature. After washing, the sections were mounted on gelatin-coated glass slides, and coverslipped in 0.01 M PBS (pH 7.4) containing 50% glycerine and 2.5% triethylenediamine, and examined using laser scanning confocal microscopy (LSCM).

### 2.10. Fluoro-Jade C Staining

We used FJC (Chemicon, Temecula, CA, USA), which can specifically stain degenerating neurons in the CNS subject to various neurotoxin insults and neurological diseases [35, 36].

Brain sections containing the hippocampus were prepared as in Section 2.9. FJC staining was carried out using the following standard procedures [35, 36]: (1) pretreatment with an alcohol-sodium hydroxide mixture. The sections were immersed in a solution containing 1% sodium hydroxide in 80% alcohol for 5 min, followed by 70% alcohol and distilled water each for 2 min. (2) Pretreatment with potassium permanganate. The sections were then transferred into a solution of 0.06% potassium permanganate for 10 min, and rinsed in distilled water for 2 min. (3) FJC staining. The sections were immersed into 0.0001% solution of FJC dye (Chemicon) dissolved in 0.1% acetic acid vehicle (pH 3.5) and stained for 10 min. (4) Post FJC treatment. The slides were washed with distilled water three times for 1 min each time and left to dry overnight in the dark at room temperature. (5) The sections were air-dried, dehydrated in ethanol, cleared in xylene, and coverslipped with DPX (distyrene, a plasticizer, and xylene). Finally, the FJC-stained sections were examined under an epifluorescence microscope or by LSCM (FluoView1000, Olympus, Tokyo, Japan). The FJC-positive staining appeared as a strong green color using the same filter system as that used for activating fluorescein.

### 2.11. Quantitative Real-Time Reverse Transcription PCR (qRT-PCR) Analysis

qRT-PCR analysis was performed for apoptosis-related genes in the hippocampus. The rat supraoptic nucleus (SON) or hippocampus was collected and total RNA was obtained from the tissues using the Trizol reagent (Invitrogen, Waltham, MA, USA) according to the manufacturer's protocol. The mRNA was then reverse transcribed to cDNA. The quantitative real-time PCR step was performed using the ABI 7901HT sequence detection system (Applied Biosystems, Foster City, CA, USA) using a Power SYBR green PCR Master Mix kit (Applied Biosystems) and the cDNA as the template. All the data were normalized to *Actb* (*β*-actin) expression.

The primers used were designed and synthesized by Takara (Dalian, China) and their sequences were as follows: *Actb*, forward primer: CACGATGGAGGGGCCGGACTCATC, reverse primer: TAAAGACCTCTATGCCAACACAGT; *Bcl2* (Bcl-2), forward primer: ATGCCTTTGTGGAACTATATGGC, reverse primer: GGTATGCACCCAGAGTGATGC; *p53*, forward primer: AAGCCCTCCAAGTGTCAGC; reverse primer: CGTCACCATCAGAGCAACG; *Bax* (Bcl-2 associated X protein), forward primer: TGAAGACAGGGGCCTTTTTG, reverse primer: AATTCGCCGGAGACACTCG; and *Casp3* (caspase-3), forward primer: ATGGAGAACAACAAAACCTCAGT; reverse primer: TTGCTCCCATGTATGGTCTTTAC.

### 2.12. Statistical Analysis

The results are expressed as the mean ± S.E.M. The data were tested for normality using the Kolmogorow–Smirnov (K-S) test. For the MWM data, the differences between the Sham-SE, Sham-EE group, IE-SE group, and IE-EE group were analyzed using two-way analysis of variance (ANOVA). For the data of FJC staining, immunofluorescent microscopy, qRT-PCR, ELISA, oxidant activity, and antioxidant activity testing, one-way ANOVA was used. When a statistically significant difference was found, Tukey's post-hoc analysis was conducted. *p* < 0.05 was considered statistically significant. The statistical analysis was performed using GraphPad Prism 9.0 software (GraphPad Inc., La Jolla, CA, USA).

## 3. Results

### 3.1. Pre-Exposure to EE Ameliorated Infrasound-Induced Learning and Memory Impairment

In this part, the effect of pre-exposure to EE on learning and memory was determined using the MWM, a hippocampus-dependent learning and memory task.

During the acquisition phase (learning testing), escape latency (latency to reach the hidden platform) was recorded for 5 consecutive days. The findings revealed that the Sham-SE rats, the Sham-EE rats, and IE-EE rats performed better in terms of escape latency than the IE-SE rats at days 3, 4, and 5, suggesting that pre-EE housing ameliorated the infrasound-induced impairment of learning (at day 3: IE factor F (1, 20) =64.7, *p* ≤ 0.001; environmental factor F (1, 20) =0.3513, *p* = 0.5600; interaction F (1, 20) =9.290, *p* = 0.0063; at day 4: IE factor F (1, 20) =54.90, *p* ≤ 0.001; environmental factor F (1, 20) =29.91, *p* ≤ 0.001; interaction F (1, 20) =15.45, *p* ≤ 0.001; at day 5: IE factor F (1, 20) =84.60, *p* ≤ 0.001; environmental factor F (1, 20) =70.85, *p* ≤ 0.001; interaction F (1, 20) =111.0, *p* ≤ 0.001. Post-hoc analysis: Sham-SE *vs*. IE-SE group: day 3: *p* ≤ 0.001, day 4: *p* ≤ 0.001, day 5: *p* ≤ 0.001; Sham-EE *vs*. IE-SE group: day 3: *p* ≤ 0.001, day 4: *p* ≤ 0.001, day 5: *p* ≤ 0.001; IE-EE *vs*. IE-SE group: day 3: *p* = 0.0784, day 4: *p* ≤ 0.001, day 5: *p* ≤ 0.001; Figure 2(a)). In addition, latency to the visible platform was also recorded at day 6. The results showed no significant differences among the three groups (IE factor F (1, 20) =0.7492, *p* = 0.3970; environmental factor F (1, 20) =0.01529, *p* = 0.9028; interaction F (1, 20) =0.01529, *p* = 0.9028; Figure 2(b)), confirming that vision was not responsible for the differences observed in escape latencies. The above results suggested that pre-exposure to EE ameliorated infrasound-induced learning deficits.

At day 6, the platform was removed and time spent in quadrant C and crossing times of the platform zone were recorded and analyzed for reference/working memory retention. The results showed that the rats in the Sham-SE, Sham-EE, and IE-EE group displayed enhanced memory retention, as demonstrated by a greater percentage of allotted time spent in quadrant C, when compared with the IE-SE rats (IE factor F (1, 20) =32.27, *p* ≤ 0.001; environmental factor F (1, 20) =9.468, *p* = 0.0059; interaction F (1, 20) =0.8964, *p* = 0.3551. Post-hoc analysis: Sham-SE *vs*. IE-SE group: *p* ≤ 0.001; Sham-EE *vs*. IE-SE group: *p* ≤ 0.001; IE-SE *vs*. IE-EE group: *p* = 0.0455; Figure 2(c)), suggesting that pre-exposure to EE relieved infrasound-induced memory impairment. The results of the crossing times of the platform zone showed similar results. The rats in the Sham-SE, Sham-EE, and IE-EE group crossed the platform zone more often than those in the IE-SE group (IE factor F (1, 20) =14.31, *p* = 0.0012; environmental factor F (1, 20) =5.990, *p* = 0.0237; interaction F (1, 20) =8.366, *p* = 0.009. Post-hoc analysis: Sham-SE *v*s. IE-SE group: *p* ≤ 0.001; Sham-EE *vs*. IE-SE group: *p* = 0.0014; IE-SE *vs*. IE-EE group: *p* = 0.0060; Figure 2(d)).

Lastly, no significant differences in swimming speed were observed among the three groups (IE factor F (1, 20) =0.052, *p* = 0.8208; environmental factor F (1, 20) =1.536, *p* = 0.2296; interaction F (1, 20) =0.608, *p* = 0.4443; Figure 2(e)), indicating that no motor deficits contributed to the above differences.

No differences were shown between the Sham-EE group and Sham-SE group in the learning and memory testing, which indicated that environmental factors had no effect on the sham rats. Thus, to highlight the mechanisms underlying the positive effect of pre-exposure to EE on the learning and memory ability under IE conditions, we only set a Sham-SE group as the control in the following mechanism experiments.

### 3.2. Pre-Exposure to EE Inhibited Inflammatory Cytokines but Enhanced Anti-Inflammatory Cytokines

In the hippocampus, accumulating evidence has demonstrated that inflammatory mediators play important roles in the impairment of learning and memory [37–40]. Accordingly, in the present study, we examined the changes of inflammatory mediators in the hippocampus.

**Figure 3:**
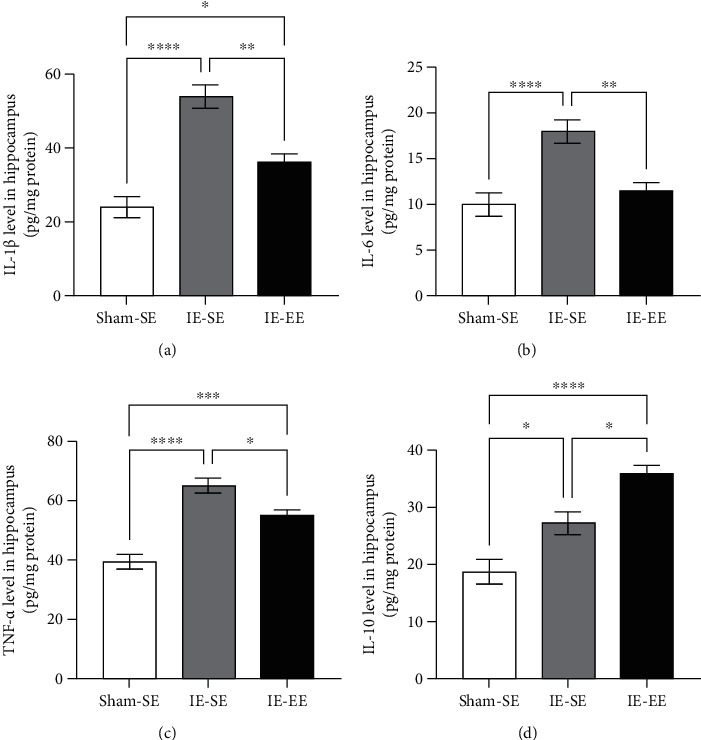
(a) The IL-1*β* level in the hippocampus in the IE-SE group was higher than that in the Sham-SE or IE-EE group. (b) The IL-6 level in the hippocampus in the IE-SE group was higher than that in the Sham-SE or IE-EE group. (c) The TNF*α* level in the hippocampus in the IE-SE group was higher than that in the Sham-SE or IE-EE group. (d) The IL-10 level in the hippocampus in the IE-SE group was higher than that in the Sham-SE group and was lower than that in the IE-EE group. ^∗^*p* < 0.05, ^∗∗^*p* < 0.01, ^∗∗∗^*p* < 0.001, ^∗∗∗∗^*p* < 0.0001. Data represent means ± SEM, *n* =6.

Compared with that in the Sham-SE group, there was an increase in IL-1*β*, IL-6, TNF-*α*, and IL-10 levels in the hippocampus of the IE-SE rats (Sham-SE *vs*. IE-SE group: IL-1*β*: *p* ≤ 0.001, IL-6: *p* ≤ 0.001, TNF-*α*: *p* ≤ 0.001, IL-10: *p* = 0.0136; Figures 3(a)–3(d)). At the same time, the levels of IL-1*β*, IL-6, and TNF-*α* in the IE-EE group were also lower than those in the IE-SE group (IE-SE *vs*. IE-EE group: IL-1*β*: *p* = 0.0010, IL-6: *p* = 0.0036, TNF-*α*: *p* = 0.0187, Figures 3(a)–3(d)). In contrast, as an anti-inflammatory cytokine, the IL-10 level in the IE-EE group exhibited an increased trend when compared with that in the IE-SE group (IE-SE *vs*. IE-EE group: IL-10: *p* = 0.0120; Figures 3(a)–3(d)), indicating that pre-EE housing skewed the IE-induced proinflammatory profile towards an anti-inflammatory profile in the hippocampus.

Taken together, these results suggested that pre-exposure to EE induced a decrease in proinflammatory cytokines and an increase in anti-inflammatory cytokines.

### 3.3. Pre-Exposure to EE Decreased Oxidative Stress in the Hippocampus

It is widely reported that oxidative stress is responsible for the learning and memory impairment in various pathological conditions [41, 42]. In this part, we detected the changes in oxidant activity in the hippocampus.

**Figure 4:**
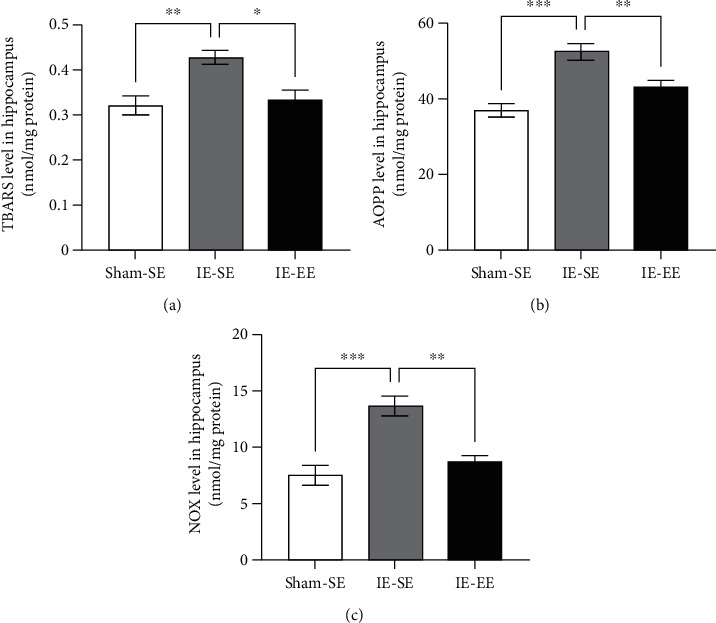
(a) The TBARS level in the hippocampus in the IE-SE group was higher than that in the Sham-SE or IE-EE group. (b) The AOPP level in the hippocampus in the IE-SE group was higher than that in the Sham-SE or IE-EE group. (c) The NOx level in the hippocampus in the IE-SE group was higher than that in the Sham-SE or IE-EE group. ^∗^*p* < 0.05, ^∗∗^*p* < 0.01, ^∗∗∗^*p* < 0.001. Data represent means ± SEM, *n* =6.

In the IE-SE group, there was a significant increase in TBARS level when compared with that in the Sham-SE group (Sham-SE *vs*. IE-SE group: *p* = 0.0048; Figure 4(a)), while the TBARS level in the IE-EE group did not differ significantly from that in the Sham-SE group and was lower than that in the IE-SE group (IE-SE *vs*. IE-EE group: *p* = 0.0121; Figure 4(a)).

A similar response was found for AOPP (Figure 4(b)). After infrasound exposure, the AOPP level in the hippocampus of the IE-SE group was significantly higher than that in the Sham-SE group (Sham-SE *vs*. IE-SE group: *p* ≤ 0.001; Figure 4(b)). Again, the IE-EE group also presented a lower AOPP level compared with that in the IE-SE group (IE-SE *vs*. IE-EE group: *p* = 0.0097; Figure 4(b)).

In this study, we also detected the changes in NOx. As expected, IE also induced a significant increase in the level of NOx compared with that in the Sham-SE group (Sham-SE *vs*. IE-SE group: *p* ≤ 0.001; Figure 4(c)). However, for the rats subjected to pre-exposure to EE and IE, the NOx level returned to basal values and showed no difference with that in the Sham-SE group (IE-SE *vs*. IE-EE group: *p* = 0.0012; Figure 4(c)).

Analysis of the markers of oxidative stress showed that exposure to infrasound caused significant oxidative stress, which could be inhibited by pre-exposure to EE.

### 3.4. Pre-Exposure to EE Enhanced Antioxidant Activity in the Hippocampus

Oxidative stress depends on the balance between antioxidant and oxidant elements. Considering that IE increased hippocampal oxidative stress, the present results also showed that IE led to a significant decrease in the principal antioxidant molecules in the hippocampus, GSH, and SOD.

**Figure 5:**
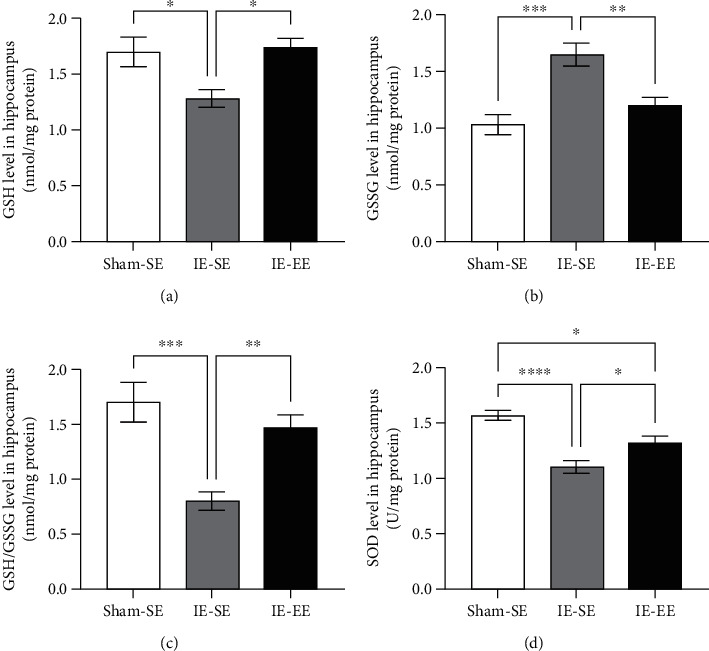
(a) The GSH level in the hippocampus in the IE-SE group was lower than that in the Sham-SE or IE-EE group. (b) The GSSG level in the hippocampus in the IE-SE group was higher than that in the Sham-SE or IE-EE group. (c) The ratio of GSH/GSSG in the hippocampus in the IE-SE group was lower than that in the Sham-SE or IE-EE group. (d) The SOD level in the hippocampus in the IE-SE group was lower than that in the Sham-SE or IE-EE group. ^∗^*p* < 0.05, ^∗∗^*p* < 0.01, ^∗∗∗^*p* < 0.001, ^∗∗∗∗^*p* < 0.0001. Data represent means ± SEM, *n* =6.

GSH was oxidized after IE, with a reduction in its hippocampal level when compared with that in the Sham-SE group (Sham-SE group *vs*. IE-SE group: *p* = 0.029, Figure 5(a)). Correspondingly, after IE, an increase in GSSG was found in the hippocampus (Sham-SE *vs*. IE-SE group: *p* ≤ 0.001; Figure 5(b)), which resulted in a decrease in the oxidized GSH/GSSG ratio in the IE-SE group (Sham-SE *vs*. IE-SE group: *p* ≤ 0.001; Figure 5(c)). However, after pre-exposure to EE, the decrease in GSH and the increase in GSSG caused by IE were inhibited (IE-SE *vs*. IE-EE group: GSH: *p* = 0.0185, GSSG: *p* = 0.0065; Figures 5(a) and 5(b)), leading to a relative higher GSH/GSSG ratio (IE-SE *vs*. IE-EE group: *p* = 0.0072; Figure 5(c)).

Another important enzyme with antioxidant activity is superoxide dismutase (SOD), which showed a significant decrease in the IE-SE group (Sham-SE *vs*. IE-SE group: *p* ≤ 0.001; Figure 5(d)). However, in the IE-EE group, the SOD activity in the hippocampus was restored during IE (IE-SE *vs*. IE-EE group: *p* = 0.0313; Figure 5(d)).

### 3.5. Pre-Exposure to EE Regulated Apoptosis-Related Molecules

Exposure to infrasound causes a significant increase in apoptosis, which also contributes to the impairment of learning and memory. P53 and Bcl-2 have been shown to regulate the apoptotic processes in opposite manners [43–45].

**Figure 6:**
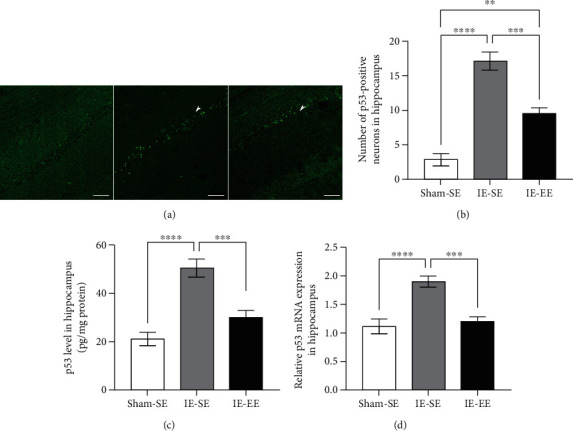
(a) P53 staining in the hippocampus in the Sham-SE, IE-SE, and IE-EE groups. (b) The number of p53-positive neurons in the hippocampus in the IE-SE group was significantly higher than that in the Sham-SE or IE-EE group. (c) The p53 protein level in the hippocampus in the IE-SE group was significantly higher than that in the Sham-SE or IE-EE group. (d) The *p53* mRNA level in the hippocampus in the IE-SE group was significantly higher than that in the Sham-SE or IE-EE group. ^∗∗^*p* < 0.01, ^∗∗∗^*p* < 0.001, ^∗∗∗∗^*p* < 0.0001. Data represents means ± SEM, *n* =6.

**Figure 7:**
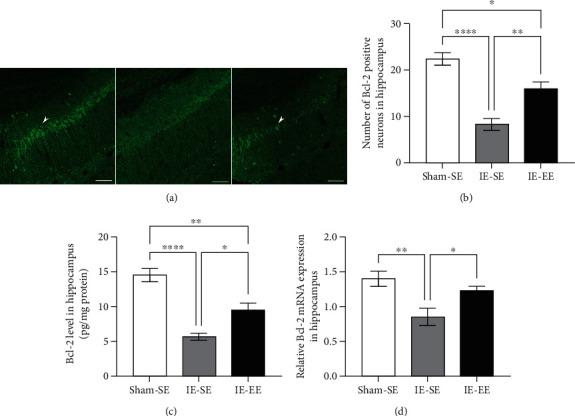
(a) Bcl-2 staining in the hippocampus in the Sham-SE, IE-SE, and IE-EE groups. (b) The number of Bcl-2-positive neurons in the hippocampus in the IE-SE group was significantly lower than that in the Sham-SE or IE-EE group. (c) The Bcl-2 protein level in the hippocampus in the IE-SE group was significantly lower than that in the Sham-SE or IE-EE group. (d) The *Bcl2* mRNA level in the hippocampus in the IE-SE group was significantly lower than that in the Sham-SE or IE-EE group. ^∗^*p* < 0.05, ^∗∗^*p* < 0.01, ^∗∗∗∗^*p* < 0.0001. Data represent means ± SEM, *n* =6.

As shown in Figure 6(a), p53-positive neurons were present in the hippocampus. In the Sham-SE group, the mean number of p53-positive neurons was about 2.83 ± 0.87 (Figures 6(a) and 6(b)). However, in the IE-SE group, the mean number of p53-positive neurons increased significantly to 17.17 ± 1.32, whereas this increase was inhibited in the IE-EE rats (9.50 ± 0.88) (Sham-SE *vs*. IE-EE group: *p* = 0.0012; IE-SE *vs*. IE-EE group: *p* ≤ 0.001 (Figures 6(a) and 6(b)). However, the number of Bcl-2-positive neurons showed the reverse change: after IE, the mean number of Bcl-2-positive neurons significantly decreased from 22.50 ± 0.87 to 8.33 ± 1.28 (Sham-SE *vs*. IE-SE group: *p* ≤ 0.001, Figures 7(a) and 7(b)). As expected, in the IE-EE group, the mean number of Bcl-2-positive neurons was partially restored to 16.00 ± 1.46, which was higher than that in the IE-SE group (IE-SE *vs*. IE-EE group: *p* = 0.0032, Figures 7(a) and 7(b)). These results suggested that EE is capable of ameliorating the IE-induced increase in p53-positive neurons and the decrease in Bcl-2-positive neurons. Moreover, the results from ELISA experiments also showed increased p53 or decreased Bcl-2 protein levels in the hippocampus after IE, and pre-exposure to EE could neutralize these changes to the p53 and Bcl-2 levels caused by IE (p53: Sham-SE *vs*. IE-EE group: *p* ≤ 0.001, Sham-SE *vs*. IE-EE group: *p* = 0.1372; IE-SE *vs*. IE-EE group: *p* ≤ 0.001; Bcl-2: Sham-SE *vs*. IE-SE group: *p* ≤ 0.001, Sham-SE *vs*. IE-EE group: *p* = 0.0019; IE-SE *vs*. IE-EE group: *p* = 0.0138; Figures 6(c) and 7(c)).

**Figure 8:**
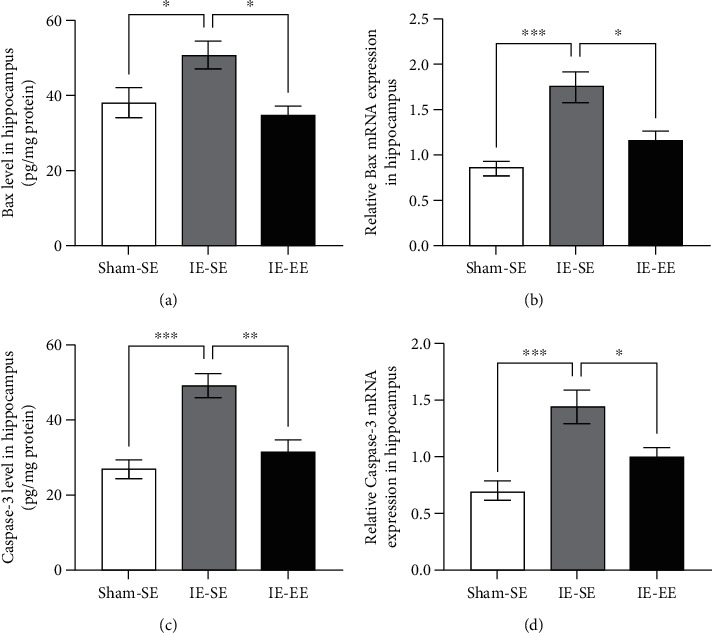
(a) The Bax level in the hippocampus in the IE-SE group was higher than that in the Sham-SE or IE-EE group. (b) The *Bax* mRNA level in the hippocampus in the IE-SE group was higher than that in the Sham-SE or IE-EE group. (c) The caspase-3 level in the hippocampus in the IE-SE group was higher than that in the Sham-SE or IE-EE group. (d) The *Casp3* mRNA level in the hippocampus in the IE-SE group was higher than that in the Sham-SE or IE-EE group. ^∗^*p* < 0.05, ^∗∗^*p* < 0.01, ^∗∗∗^*p* < 0.001. Data represent means ± SEM, *n* =6.

Previous studies have shown that Bcl-2 reduced apoptosis by influencing Bax and caspase-3 [28]. Therefore, we also detected the changes in Bax and caspase-3 in the hippocampus using ELISA. As expected, infrasound exposure increased the levels of Bax and caspase-3 (Bax: Sham-SE *vs*. IE-SE group: *p* = 0.0426, caspase-3: Sham-SE *vs*. IE-SE group: *p* ≤ 0.001; Figures 8(a) and 8(c)) and pre-exposure to EE could inhibit the increase in the levels of Bax and caspase-3 (Bax: IE-SE *vs*. IE-EE group: *p* = 0.0108, caspase-3: IE-SE *vs*. IE-EE group: *p* = 0.0026; Figures 8(a) and 8(c)).

qRT-PCR showed similar results. IE induced increases in *P53*, *Bax*, and *Casp*3 expression, but a decrease in *Bcl2* expression in the hippocampus, which could be blocked by pre-EE housing (*p53*: Sham-SE *vs*. IE-SE group: *p* ≤ 0.001, IE-SE *vs*. IE-EE group: *p* ≤ 0.001; *Bcl2*: Sham-SE *vs*. IE-SE group: *p* = 0.0048, IE-SE *vs*. IE-EE group: *p* = 0.0461; *Bax*: Sham-SE *vs*. IE-SE group: *p* ≤ 0.001, IE-SE *vs*. IE-EE group: *p* = 0.0105; *casp*3: Sham-SE *vs*. IE-SE group: *p* ≤ 0.001, IE-SE *vs*. IE-EE group: *p* = 0.0303; Figures 6(d), 7(d), and 8(b) and 8(d)).

Thus, pre-exposure to EE exhibited antiapoptosis activity under infrasound exposure conditions by affecting the levels of p53 and the Bcl-2/Bax/caspase-3 signaling pathway.

### 3.6. Pre-Exposure to EE Inhibited the Apoptotic Response to IE

**Figure 9:**
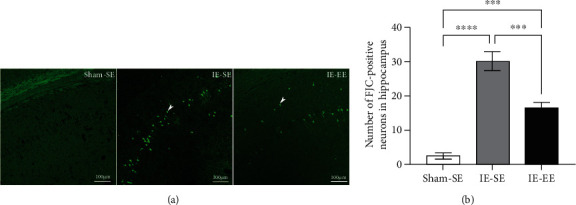
(a) FJC staining in the hippocampus in the Sham-SE, IE-SE, and IE-EE groups. (b) The number of FJC-positive neurons in the hippocampus in the IE-SE group was significantly higher than that in the Sham-SE or IE-EE group. ^∗∗∗^*p* < 0.001, ^∗∗∗∗^*p* < 0.0001. Data represent means ± SEM, *n* =6.

In the present study, apoptotic neurons were stained using FJC dye in the hippocampus. As shown in Figure 9, the FJC-positive cells were clearly observed in the hippocampal pyramidal layer and granular cells of the dentate gyrus. In the Sham-SE group, no FJC-positive neurons were detected in the hippocampus (Figures 9(a) and 9(b)). However, in the IE-SE group, the mean number of FJC-positive neurons dramatically increased to 30.17 ± 2.74 (Sham-SE *vs*. IE-SE group: *p* ≤ 0.001; Figures 9(a) and 9(b)). Furthermore, in the IE-EE group, the mean number of FJC-positive neurons was 16.50 ± 1.61, which was less than that in the IE-SE group (IE-SE *vs*. IE-EE group: *p* ≤ 0.001; Figures 9(a) and 9(b)). These results suggested that infrasound enhanced apoptosis in the hippocampus, which could be inhibited by pre-exposure to EE.

## 4. Discussion

The results of the present study provided evidence that 30 days of EE before infrasound treatment protects learning and memory, as indicated by the results of the MWM test. Animals pretreated with EE has better prognosis following infrasound exposure. This is likely achieved by enhancing antioxidant, anti-inflammatory, and antiapoptosis capacities.

In the present study, we used the MWM to evaluate the learning and memory ability. The MWM is widely used to assess hippocampus-dependent spatial learning and memory and is closely related to hippocampal long-term potentiation (LTP) [46–48]. In the present study, the increased escape latency, less time in target quadrant C, and reduced target crossing times after IE indicated impaired learning and memory, which were consistent with the results of previous studies [7, 11]. Our study also revealed that pre-exposure to EE was capable of counteracting the infrasound-induced impairment of learning and memory. We noticed that the rats swam at about the same speed in the pool among the groups, suggesting that no locomotor factor disturbed their performance in the MWM. In addition, land-based locomotor impairment is not related to swimming speed, which also accounts for the independence of learning and memory performance in the MWM from locomotor effects.

Neuroinflammation plays important roles in learning and memory deficits under pathological conditions [49–51]. In the present study, we demonstrated that after infrasound exposure, the levels of IL-1*β*, IL-6, and TNF-*α* increased significantly, which were consistent with the results of previous studies [11, 12]. As a proinflammatory cytokine, an increased IL-1*β* level inhibits LTP in the hippocampus by affecting Ca^2+^ conductance through N-methyl-D-aspartate receptors (NMDARs) [52]. In increased IL-6 level also impairs the LTP by decreasing extracellular regulated kinase (ERK)1/2 activation in the hippocampus [53]. Moreover, increased hippocampal TNF*α* concentrations can block glutamate transporter activity and promote glutamate neurotoxicity, eventually leading to an impaired LTP [54]. Therefore, our results indicated that IE might impair the learning and memory ability by increasing these proinflammatory cytokines. By contrast, IL-10 is the most important anti-inflammatory cytokine, which counteracts the damage caused by excessive inflammation. In this study, we found that infrasound exposure induced an obvious increase in anti-inflammatory cytokine (IL-10). By acting on the IL-10 receptor in neurons, IL-10 facilitates the LTP via regulation of GABA_B_ synaptic transmission, thereby increasing the learning and memory ability [55]. The increase in IL-10 might be the result of a self-protection mechanism against infrasound exposure. In addition, the present study found that EE pre-exposure counteracted the IE-induced change in inflammatory mediators in the hippocampus, suggesting a positive effect of EE on the learning and memory ability under IE conditions. In fact, it has been reported that EE affects cytokines, various immune components, and glial cells under various pathological conditions [24, 56–58]. The antineuroinflammatory effect of EE might be achieved through several immune pathways [59, 60]: (i) increased migration of macrophages into the CNS and enhancement of their regulatory effects on microglia; (ii) upregulation of mitogen-activated protein kinase (MAPK) phosphatase-1 (MKP-1), which exhibits negatively regulatory roles in proinflammatory macrophage MAPK activation; and (iii) modulation of hippocampal T cells, which are responsible for the modulation of microglia. Accordingly, we will investigate the effect of EE pre-exposure on these immune pathways under infrasound exposure conditions in a future study.

The present study also indicated that TBARS and AOPP (markers of oxidative stress) increased after infrasound exposure and EE pre-exposure could reduce these increases in oxidative stress. Past studies have shown that increased learning and memory performance in rats is related to a decrease in hippocampal oxidative stress [61–63]. For example, amyloid *β* (A*β*) can induce spatial learning and memory impairment that can be inhibited by blocking the increase in oxidative stress [64]. Therefore, in the present study, we propose the improvement of learning and memory caused by EE pre-exposure function by decreasing oxidative stress.

Under physiological conditions, there is a balance between oxidative stress and the antioxidant system. In the present study, infrasound reduced the amount of GSH in the hippocampus and increased the amount of GSSG, with a consequent reduction of the GSH/GSSG ratio, indicating lower scavenging capacity of the glutathione system in the hippocampus. Besides the reduced GSH/GSSG ratio, the SOD level was also suppressed by infrasound. SOD can catalyze superoxide anions into oxygen and hydrogen peroxide [65], which has an important protective role against the effects of reactive oxygen species (ROS). These results agree with some previous studies [9, 11]. In this study, we found that pre-exposure to EE was effective to block the infrasound-induced decrease in the GSH/GSSG ratio and SOD level in the hippocampus, indicating that EE pre-exposure improved the antioxidant system. Oxidative stress increases the production of ROS in the brain, which plays a positive role in modulating the production of proinflammatory mediators by preventing MAPK and nuclear factor kappa B (NF-*κ*B) activation in microglia cells [66]. Therefore, we concluded that EE pre-exposure skews the infrasound-induced increase in oxidative stress and decreased the antioxidant system, resulting in a decrease in proinflammatory cytokines.

Infrasound induces neuronal apoptosis, which is also associated with learning and memory deficiency [67–69]. The present study observed that pre-exposure to EE inhibited neuronal apoptosis in the hippocampus, suggesting antiapoptosis as one of the mechanisms underlying EE pre-exposure blockade of infrasound-induced impairment of learning and memory. Our results also showed that pre-exposure to EE could inhibit the infrasound-induced increase in p53 and decrease in Bcl-2. The p53 protein mainly regulates cell-cycle arrest, senescence, and apoptosis [70, 71]. Clearly, stress or trauma can lead to p53 activation [72]. Therefore, as a background stressor, infrasound activates p53, which results in increased apoptosis in the hippocampus. Unlike p53, Bcl-2 is a small intracellular nonglycosylated protein that inhibits the apoptotic pathway when overexpressed in cells [73]. Our study also demonstrated a significant inverse relationship between Bcl-2 and p53 protein levels in the hippocampus after infrasound treatment. This finding is in line with previous studies about relationship between Bcl-2 and p53 protein levels [74]. There is also the crosstalk between Bcl-2/p53 and inflammatory mediators. For example, activation NF-*κ*B/p53 signaling can enhance the increase in inflammatory mediators [75], while Bcl-2 exerts an anti-inflammatory function through inhibition of NF-*κ*B [76].

This study had some limitation: (1) We assessed the learning and memory ability, detected cytokine levels, oxidative stress, antioxidant activity, apoptosis-related molecules, and apoptosis only at a single time point (i.e., after 14 days of infrasound exposure). In a future study, we will measure the above indices at different exposure times to determine the temporal effect of pre-exposure to EE. (2) In the mechanism experiments, we only set a Sham-SE group as the control. Although there were no differences between the Sham-EE group and Sham-SE group in the outcome of the MWM, a Sham-EE group should have been included in the mechanism experiments. Nonetheless, the primary aim of mechanism experiments was to identify the mechanisms underlying the effect of pre-exposure to EE on the learning and memory ability under IE conditions. The evidence from the present experimental design was sufficient to determine the possible mechanisms underlying this process. In addition, past studies also revealed no differences in the level of inflammatory factors between the Sham-EE group and Sham-SE group [77, 78]. (3) Besides the enriched and novel environment, EE housing is also accompanied by social enrichment and intermittent physical exercise in animal experiments, which are known to promote neuroprotection [79]. Therefore, we did not rule out the role of social enrichment or physical exercise in the pre-exposure to EE-induced improvement of learning and memory in the present study. A recent study showed that EE and physical exercise have better neuroprotective effects than social enrichment in memory deficits related to amyloid *β* (A*β*) neurotoxicity in an Alzheimer's (AD) disease model [79].

In conclusion, the results of the present study showed that pre-exposure to EE is effective to ameliorate the learning and memory impairment caused by infrasound exposure. This process is related to a decrease in proinflammatory cytokines, oxidative stress, and apoptosis, and an increase in anti-inflammatory cytokines and antioxidant activity. The exact molecular mechanism will be explored in a future study. Therefore, these results supported the view that pre-exposure to EE could be a viable training mechanism to improve resilience against the consequences of infrasound.

## Data Availability

All data can be available on the inquiry for the corresponding authors.
